# Integrated application of meat waste-derived organic fertilizer and chemical fertilizer in sugarcane cultivation

**DOI:** 10.1371/journal.pone.0352193

**Published:** 2026-06-26

**Authors:** Aselefech Sorsa Wotango, Feredegn Dagnaw, Mulatu Kassie Birhanu, Asnake Waltenigus Ewnetu, Tadesse Negi, Mesfin Tafesse Gemeda, Getachew Adam Workneh

**Affiliations:** 1 Department of Industrial Chemistry, College of Natural and Applied Sciences, Addis Ababa Science and Technology University, Addis Ababa, Ethiopia; 2 Biotechnology and Bioprocess Center of Excellence, Addis Ababa Science and Technology University, Addis Ababa, Ethiopia; 3 Sustainable Energy Center of Excellence, Addis Ababa Science and Technology University, Addis Ababa, Ethiopia; 4 Wonji-Shoa Sugar Factory, Wonji, Ethiopia; 5 Manufacturing Industry Development Institute, Addis Ababa, Ethiopia; 6 Ethiopian Sugar Industry Group Research and Training Division, Wonji, Ethiopia; Veracruzana University: Universidad Veracruzana, MEXICO

## Abstract

Slaughterhouse waste is a major source of environmental pollution. However, its rich organic content holds considerable potential for enhancing soil fertility. Herein, slaughterhouses meat waste was used to produce organic fertilizer (OF), and its effect on sugarcane growth, productivity and soil fertility were evaluated. The decomposition of the collected meat waste was carried out in a controlled fermentation pit. Initially, the sample was treated by sulfuric acid (H_2_SO_4_), followed by the addition of potassium hydroxide (KOH) and dipotassium phosphate (K_2_HPO_4_). The pH of the medium was then adjusted to neutral conditions, after that a bacterial inoculum was introduced to facilitate further biodegradation of the solid waste. The prepared organic fertilizer was characterized for its chemical compositions, physicochemical properties and microbial diversity. The chemical compositions analyses revealed that potentially toxic elements such as mercury (Hg), lead (Pb), cadmium (Cd) and arsenic (As) were detected at concentration of <0.002 mg/kg, 0.411 mg/kg, 0.004 mg/kg and 0.095 mg/kg, respectively, indicating their levels in the fertilizer were very low. The total organic carbon content was determined to be 13.90% that is significantly higher than that of most soil, where organic carbon levels typically fall below 2%. MALDI-TOF (Matrix-Assisted Laser Desorption/Ionization Time-of-Flight) mass spectrometry was used to identify culturable bacterial isolates obtained from the fermented organic fertilizer prior to soil application. A total of 20 morphological distinct isolates were analyzed, of which Lysinibacillus sphaericus was the most frequently detected species, followed by Bacillus simplex, Bacillus cereus, Kocuria varians, and Microascus sp. Identification was accepted based on MALDI-TOF score thresholds (≥2.0 species-level and 1.7–1.99 for genus level identification). *Lysinibacillus sphaericus* aids nitrogen-fixation, plant growth, and decomposing organic matter, all of which enhance soil fertility and plant health. Additionally, other species such as *Bacillus simplex, Bacillus cereus, Kocuria varians*, and *Microascuswere* contribute to phosphate solubilization and pathogen suppression. The organic ferilizer was tested alone and in combination with the conventional chemical fertilizer in a pot experiment for sugarcane. The results showed that applying 5 tonnes ha^-1^ of organic fertilizer together with 50% of the recommended chemical fertilizer performed comparable to that of 100% recommended chemical fertilizer alone. In addition, combined organic fertilizer and recommended chemical fertilizer resulted in a significantly higher juice phosphate content compared to the sole application of 100% recommended chemical fertilizer, likely due to the contribution of the organic fertilizer. This finding suggests that halving chemical fertilizer use by supplementing with organic alternatives could offer environmental benefits while maintaining productivity.

## 1. Introduction

Slaughterhouses occupy an essential place in the livestock-to-meat production process and are serving as a critical link in the food supply chain. In addition to meat, they produce valuable byproducts such as hides and skins, which are essential raw materials for leather industries. However, the meat production process also generates a significant amount of waste that could severely pollute the environment unless managed properly. In many developing countries, the lack of adequate access for waste processing technologies, and the increasing cost of treatment and removal, makes slaughterhouses waste a major environmental concern [1–3].

Conversion of slaughterhouses waste into organic fertilizer addresses environmental challenges due to improper disposal while promoting efficient resource utilization with sustainable and eco-friendly options. In addition, composted meat waste is rich in essential nutrients that could greatly help to improve the physical, chemical and biological properties of soil. It improves the structure of soil and makes space for air, promotes good root growth, adjusts soil pH, improves cation exchange capacity, enhances soil porosity and permeability [4,5]. Apart from that, composted meat waste could increase microbial community biomass and metabolic vigor in the soil rhizosphere and inhibit rot and pathogens [6]. Additionally, converting meat waste into organic fertilizer reduces the environmental burden associated with treating slaughterhouse effluents and minimizes the need for chemical fertilizers, thereby lowering agricultural cost and lessening pollution from chemical fertilizers [7].

Several studies have been investigated on the effects of slaugherhouse meat waste-derived organic fertilizer for various crops. Roy et al. examined an organic fertilizer produced from a bovine-blood-rumen-digesta-mixture (BBRDM) for tomato cultivation. Their results showed that tomato plants treated with BBRDM produced yields 48% higher than those treated with a combination of diammonium phosphate (DAP) and potash [8]. Similarly, the application of 6 g BBRDM per kg of soil in bell pepper cultivation resulted in a two-fold increase in yield compared to fertilize contained N/K/P: 10:26:26 plus urea treatment [9]. In other study, the agronomic performance of organic fertilizer derived from hull and horn flour and blood fluor residue was compared to conventional urea in the cultivation of mombasa grass. The finding indicated that organic fertilizer application enhanced several growth parameters, including leaf area, number of tillers, crude proteins and forage mass [10]. Despite these promising results, comprehensive evaluations of organic fertilizer effects across a wider range of plant species is limited. In Ethiopia, sugarcane makes a significant contribution to the local sugar supply and also holds a share in the international market [11,12]. Sugarcane cultivation typically requires chemical fertilizer to achieve satisfactory yield, however, these fertilizers are largely imported and incur high costs. The combined application of organic and chemical fertilizers in sugarcane cultivation significantly improved cane yield [13–15]. In Ethiopia, the combined application of vinasse-filtercake composte with urea as a nitrogen source has been investigated for improving sugarcne productivity at the Metehara plantation site. Nevertheless, to the best of our knowledge, no studies have reported sugarcane cultivation in Ethiopian soils using organic fertilizer derived from local slaugherhouses waste.

In the study, meat waste collected from slaughterhouses was processed using a controlled fermentation procedure to decompose the larger organic matter into smaller and more manageable forms. This decomposition transformed the raw waste material into nutrient-rich compost and made it suitable for use as an organic fertilizer. In order to assess its effectiveness, the composted fertilizer was applied in sugarcane cultivation, both with and without chemical fertilizer. The assessment was focused on sugarcane growth and productivity as well as the changes in the soil’s physicochemical properties.

## 2. Experimental section

### 2.1. Preparation of organic fertilizer

A total of 75 kg freshly processed cattle waste (meat & hide) was collected from legally registered local slaughterhouses. The experiment was conducted in accordance with the ethical standards of Addis Ababa Science and Technology University. Ethical clearance for the research was obtained from the Institutional Ethical Approval Board under approval number AASTU-IEAB Minute 021/22. The local slaughter houses described in the study are small, community-based facilities located within condominium residential compounds and commonly referred to as communal houses. They are used solely for immediate animal slaughter. Liquid waste generated at these facilities is discharged through the designated waste drainage system, whereas solid waste must be managed and disposed of by the individuals conducting the slaughter. Animals are slaughtered exclusively for food purpose and are not kept on-site prior to slaughter. The collected waste sample was deposited into a fermentation pit (1 m × 1 m) for processing at a temperature of 29 ± 4 ºC. To initiate the breakdown of organic matter, 2% sulfuric acid (80% H_2_SO_4_) was added and allowed to act for a week [16,17]. After the acid treatment, potassium hydroxide (KOH) and dipotassium phosphate (K_2_HPO_4_) were added to neutralize the pH, creating a favorable environment for microbial activity. To introduce native microbial communities, soil was collected from areas surrounding the slaughterhouse that were not exposed to animal waste, by-products, and the waste derange system. The soil had a moisture content of 22.58%, and a texture composed of 9% sand, 13% silt and 78% clay. It was incorporated into the fermentation pit at 20% on a dry weight basis without any pretreatment [18].

To further accelerate the decomposition process, a sample of the mixture was taken to the Biotechnology laboratory at Addis Ababa Science and Technology University. Previously studied bacterium consortium, *Klebsiella oxytoca* (MT104573.1) and *Lysinibacillus macrolides* (MN932110.1) were aseptically scaled up in seed culture (100 mL) for laboratory inoculum preparation [19–21]. Each strain was cultured in a 1 L Elernmenry flask containing 500 mL of freshly prepared sterile media and incubated for 24–72 h under continuous stirring. The cell density reached 1.4 ± 0.5 × 10^8^ CFU/mL for *K. oxytoca* and 2.7 ± 0.25 × 10^8^ CFU/mL for *L. macrolides*. Subsequently, 500 mL of each bacterial culture was simultaneously reintroduced into the fermentation pit as a 5% (v/v) inoculum for each strain, resulting in total microbial load of around 3.50 × 10^7^ CFU/mL of *K. oxytoca* and 2.25 × 10^8^ CFU/mL for *L. macrolides*. The pit content was stirred daily for 10–15 minutes to ensure homogeneity and adequate oxygenation. Upon complete decomposition of the organic matter, the resulting liquid product was collected for characterization and application. All the analyses were conducted in triplicate to ensure reproducibility.

### 2.2. Physicochemical and chemical analyses

The physicochemical and chemical analyses were conducted in accordance with standard set by the Ethiopian Standard (ES), International Organization for Standard (ISO), Environmental Protection Agency (EPA), Association of Official Analytical Chemists (AOAC) and BCTL/SOP guideline. Inductively coupled plasma mass spectroscopy (ICP-MS system model 7900, Agilent, Palo Alto, CA, USA) and inductively coupled plasma optical emission spectroscopy (ICP-OES) were employed for the analyses. All analyses were carried out at the Ethiopian Conformity Assessment Enterprise (ECAE) in Addis Ababa, Ethiopia.

### 2.3. Microbial analysis

A total of 1000 mL of fermented organic fertilizer was collected from three depths (top, middle, and bottom) of the digestion pit using sterile, autoclaved reagent bottles and pooled to obtain a representative sample. Samples were stored at 4 °C and processed within 24 h [22]. Serial dilutions were prepared by transferring 1 mL of sample into 9 mL of sterile distilled water to obtain a 10 ⁻ ¹ dilution, followed by sequential dilution up to 10 ⁻ ⁵ [23]. Aliquots (100 µL) from each dilution were plated in triplicate to enable estimation of culturable bacterial presence and isolate recovery.

### 2.4. Culture media preparation and microbial isolation

Microbial isolation was performed using Nutrient agar composed of (g L^-1^): Peptone (5.0), Beef extract (3.0), Sodium chloride (5.0), Agar (15.0). A total of 2.6 g of the medium was dissolved in 100 mL of distilled water, autoclaved at 121 ºC for 15 min, cooled, and poured under aseptic conditions. Plates were incubated at 28 °C for 48 h, after which colony-forming units (CFUs) were visually assessed and morphologically distinct colonies were selected for purification [24,25].

### 2.5. Purification and morphologically characterization of isolates

Distinct colonies differing in size, shape, color, margin and elevation were repeatedly sub-cultured to obtain pure isolates [25]. In total, 20 pure isolates were obtained and preserved for downstream identification. Colony morphology was recorded to ensure representative sampling of the dominant culturable bacteria.

### 2.6. Microbial identification using MALDI-TOF mass spectrometry

A total of 20 purified isolates were analyzed using MALDI-TOF mass spectrometry following a standard ethanol-formic acid extraction protocol [26]. Spectra were acquired using a BRUKER MALDI-TOF and matched against the manufacturer’s reference database. The high-resolution spectral profiles enabled precise identification of microbial species [27]. Identification reliability was interpreted using established score thresholds: scores ≥ 2.0 indicated species-level identification, score of 1.7–1.99: indicated probable genus-level identification, and scores < 1.7 indicated unreliable identification. Score thresholds were established based on reported studies to ensure the reliability of the identification process [28]. Quality control included duplicate spectral acquisition per isolate and exclusion of spectra with poor signal-to-noise ratios. Relative frequency of detected taxa was calculated based on the number of isolates identified per species.

### 2.7. Application of the prepared fertilizer into sugarcane

The pot experiment was conducted in a greenhouse during the cropping season, using half barrels as pots, with each half barrel considered one experimental unit. The pot size was 29 cm radius x 38 cm height (3.14*0.29*0.29*0.38 = 0.1 m^3^). A total of 2.1 m^3^ soil sample at the depth of 0–30 cm was collected from a well-drained field of Wonji Sugarcane Plantation to fill individual pots. NCO-334 sugarcane variety which is widely grown in Ethiopia Sugar Industry and the dominant sugarcane variety at Wonji Shoa Sugar State was used as a test crop and two buds setts were planted in each pot. Sugarcane management practice, including planting, weeding and watering were carried out according to the crop requirements and the crop was harvested ten months after planting. At harvest, measurements were taken for plant height, leaf length, leaf width, number of internode, internode diameter, stalk weight (cane yield). Juice quality parameters, including juice phosphate, brix, pol, purity and recoverable sugar were also recorded. The samples were analyzed at Wonji Research and Training Laboratory. Sugarcane juice quality was measured based on the procedure described by Kassa [29]. There were eleven treatments arranged in randomized complete block design (RCBD) with three replications. Spacing between pots were 1.45 m and between replication was 1.45 m. The recommended chemical fertilizer rate was used as the reference (control) treatment, as it represents the standard fertilizer application commonly practiced in local sugarcane cultivation. Treatment combinations are presented in Table 1.

**Table 1:** Treatment formulas.

SN	Treatments	Remark
1	Recommended chemical fertilizer rate (RCF)	152 (kg ha^-1^) NPS at planting + 141 (kg ha^-1^) urea at 2.5 months
2	Conventional Urea (Old Recommendation)	200 (kg ha^-1^) urea at 2.5 months
3	OF alone at 2.5 (tonnes ha^-1^)	All at planting
4	OF alone at 5 (tonnes ha^-1^)	All at planting
5	OF alone at 7.5 (tonnes ha^-1^)	All at planting
6	25% of RCF + 2.5 (tonnes ha^-1^) OF	All at planting + urea at 2.5 months
7	50% of RCF + 2.5 (tonnes ha^-1^) OF	All at planting + urea at 2.5 months
8	25% of RCF + 5 (tonnes ha^-1^) OF	All at planting + urea at 2.5 months
9	50% of RCF + 5 (tonnes ha^-1^) OF	All at planting + urea at 2.5 months
10	25% of RCF + 7.5 (tonnes ha^-1^) OF	All at planting + urea at 2.5 months
11	50% of RCF + 7.5 (tonnes ha^-1^) OF	All at planting + urea at 2.5 months

**NPS (19 N + 38 P**_**2**_**O**_**5**_ **+ 7 S)**

### 2.8. Soil sample analyses

Soil samples from 0–30 cm depth were collected before treatment application and from each pot after harvest. The soil samples were analyzed at Wonji Research and Training Laboratory. The soil sample analyses were made for texture, pH, electrical conductivity, organic carbon, total nitrogen (N) and available phosphorus (P). Available P determination was determined following the reported procedure [30]. Soil particle size distribution (soil texture) was analyzed using the Bouyoucos hydrometer method and the soil textural class was assigned according to the USAD textural triangle.

### 2.9. Data analyses

Agronomic data was subjected to ANOVA using appropriate computer software (SAS 2002) and the least significant different test (p < 0.05) was used to compare means. Descriptive statistics and different rating scales were used to interpret soil and leaf analyses results. The soil results were interpreted using the rating scale of Landon [31]. The nitrogen and phosphorus fertilizer requirements of the soil were determined in accordance with the fertilizer recommendation guideline [32]. The leaf nutrient concentration analyses results were interpreted using critical values and optimum ranges defined by McCray and Mylavarapu [33].

## 3. Results and discussion

### 3.1. Characterization of the organic fertilizer

#### 3.1.1. Physicochemical properties of the organic fertilizer.

Physicochemical properties of the prepared OF are presented in Table 2. The TDS content was 7.65% and the measured pH was 7.65, indicating a slightly alkaline nature that could be beneficial for acidic soil as it could help to neutralize soil acidity. Alkaline conditions also enhance organic matter decomposition and aid in suppression of pathogens [3]. A comparable pH value of 7.5 has been reported for organic fertilizer derived from bovine-blood-rumen-digesta-mixture [9]. Electrical conductivity measurements serve as an indicator of total quantities of soluble salts present in the material. The electrical conductivity (EC) of the fertilizer was found to be 0.14 dS/m which suggests that the fertilizer has a low level of soluble salts and minimal risk of increasing soil salinity. Nevertheless, previous studies highlighted the potential of EC in enhancing nitrogen uptake efficiency in sugarcane production [34].

**Table 2:** Physiochemical properties of the prepared organic fertilizer.

Parameters	Test method	Value
pH	ES 402:2O55	7.65
TDS (Total Dissolved Solid) (% by mass)	ES 609:2001	6.29
Electrical conductivity (EC) (dS/m)	ISO 1 1 265: 1 994	0.14
Total organic carbon (% by mass)	ES 7062:2023	13.90
Ash content (% by mass)	ES ISO 2171:2022	0.89

The total organic carbon content was 13.90%, while the ash content was 0.89%. Organic carbon plays a crucial role in regulating soil physical, chemical and biological functions. It improves soil structure and enhances nutrient availability for plants and also promotes microbial activities [35]. However, due to environmental factors and poor farm management practice, the organic carbon content of soils is often very low. The soil used for planting sugarcane contained about 1.5% organic carbon. Therefore, the prepared fertilizer could increase the soil’s organic carbon, which would be beneficial for sugarcane growth.

#### 3.1.2. Chemical composition of the organic fertilizer.

The chemical composition of the prepared OF is summarized in Table 3. The analyses were conducted to determine the presence of essential and trace minerals. Toxic elements residue are available both in organic and chemical fertilizers, polluting agricultural soil and adversely affecting plant life and human health [36]. Hg, Pb, Cd and As are considered the most toxic elements depending on the exposure time and their amount [37]. The permissible value of these elements in commercial organic fertilizer were set ≤ 2 mg/kg, ≤ 50 mg/kg, ≤ 3 mg/kg and ≤ 15 mg/kg for Hg, Pb, Cd and As, respectively [38]. The prepared fertilizer revealed that Hg (<0.002 mg/kg), Pb (0.411 mg/kg), Cd (0.004 mg/kg) and As (0.095 mg/kg) indicating that very low level of these elements in the fertilizer. This is essential for ensuring the safety of both crops and the environment. The presence of organic matter in soil further reduces metal uptake by plants due to the formation of metal-organic matter complex [39].

**Table 3:** Chemical composition analyses results of the prepared organic fertilizer.

N/S	Characteristics tested	Test method /Specification	Value
1	Nitrogen Content (as N) (% by mass)	BCTL/SOP/M014.01	0.21
2	Phosphorus (as P) (% by mass)	BCTL/SOP/iVI052.u1	0.113
3	Sulphur (as SO4^2-^ equivalent) (% by mass)	BCTL/SOP/M016.01	0.45
4	Chloride (as Cl^-^) (mg/L)	EPA 300.1	215.7
5	Potassium (as K) (% by mass)	ICP-OES	0.63
6	Manganese (as Mn) (% by mass)	ICP-OES	0.01
7	Sodium (as Na) (% by mass)	ICP-OES	0.20
8	Magnesium (as Mg) (% by mass)	ICP-OES	0.06
9	Boron (as B) (mg/kg)	ICP-OES	17.0
10	Silicon (as Si) (% by mass)	ICP-OES	0.01
11	Calcium (as Ca) (% by mass)	AOAC 2015.01 ICP-MS	0.15
12	Copper (as Cu) (mg/kg)	AOAC 2015.01 ICP-MS	2.198
13	Zinc (as Zn) (mg/kg)	AOAC 2015.01 ICP-MS	4.604
14	Iron (as Fe) (mg/kg)	AOAC 2015.01 ICP-MS	<0.04
15	Arsenic (as As) (mg/kg)	AOAC 2015.01 ICP-MS	0.095
16	Cadmium (as Cd) (mg/kg)	AOAC 2015.01 ICP-MS	0.004
17	Lead (as Pb) (mg/kg)	AOAC 2015.01 ICP-MS	0.411
18	Selenium (as Se) (mg/kg)	AOAC 2015.01 ICP-MS	0.002
19	Mercury (as Hg) (mg/kg)	AOAC 2015.01 ICP-MS	<0.002
20	Nitrate (as NO_3_^-^) (mg/L)	EPA 300.1	40.43

Nitrogen (N), phosphorus (P), and potassium (K) are essential macronutrients and key limiting factors in plant growth [40,41]. These elements also constitute the main components of conventional chemical fertilizers. However, the prepared fertilizer exhibited relatively low concentration of total nitrogen (0.21%) and available phosphorus (0.113%). This low nutrient content may be attributed to the addition of soil during the fermentation process, which likely dilutes the concentration of these elements. Additionally, nitrogen loss may have occurred from the release of ammonia (NH_3_) gas during meat decomposition, which make up approximately 99% of odorous compounds [42]. In contrast, the potassium (0.63%) content was relatively higher possibly due to the addition of potassium containing compounds such as KOH and K_2_HPO_4_ during preparation.

The chloride content of the prepared fertilizer  was 215.7 mg/L, the highest among the analyzed constituents. This elevated level may be attributed to the water source used during the preparation process. Study on sugarcane cultivation has reported using irrigation water with varying chloride concentrations, ranging from 60–720 mg/L tube well water and 0.67–180 mg/L canal water [43].

#### 3.1.3. Microbial communities of the organic fertilizer.

**Fig 1:**
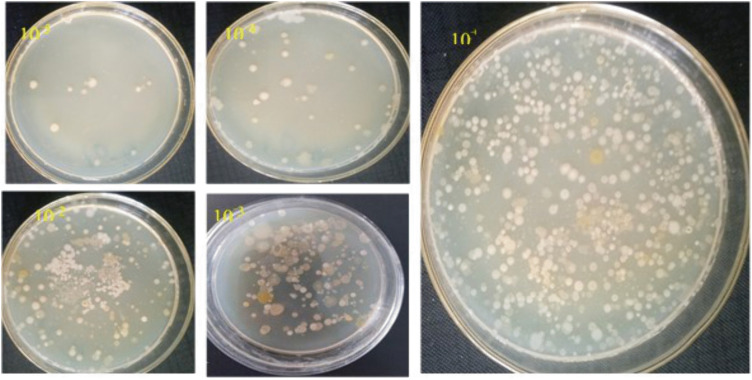
Bacterial colonies isolated from the prepared fertilizer.

Microbial identification through MALDI-TOF mass spectrometry has emerged as an effective and precise method for characterizing microbial isolates, providing valuable insights into their roles in organic fertilizer applications. In the study, several bacterial isolates were identified like Lysinibacillus *sphaericus* being the most prevalent among the isolates. This isolate is recognized for its nitrogen-fixing abilities, production of plant growth-promoting substances, and contribution to organic matter decomposition, all of which enhance soil fertility and plant health [44]. Additionally, other isolates such as *Bacillus simplex*, *Bacillus cereus*, *Kocuria varians*, and *Microascus sp.* were identified, each contributing uniquely through phosphate solubilization, pathogen suppression, and nutrient mobilization [45]. The diversity of these bacterial species underscores the significance of microbial communities in improving soil health and promoting sustainable agriculture [46]. Moreover, the complementary roles of these species suggest potential synergistic effects in organic fertilizer formulations, where combining nitrogen-fixing bacteria like *L. sphaericus with* phosphate-solubilizing *B. simplex* could provide a holistic approach to enhancing soil fertility, while the pathogen-suppressing capabilities of *B. cereus* further support plant growth [47]. They achieve this through various processes, including nitrogen fixation, phosphate solubilization, organic matter decomposition, and pathogen suppression. Their diverse functions underscore their potential in sustainable agriculture [47]. The microbial analysis result is shown in Fig 1.

**Fig 2:**
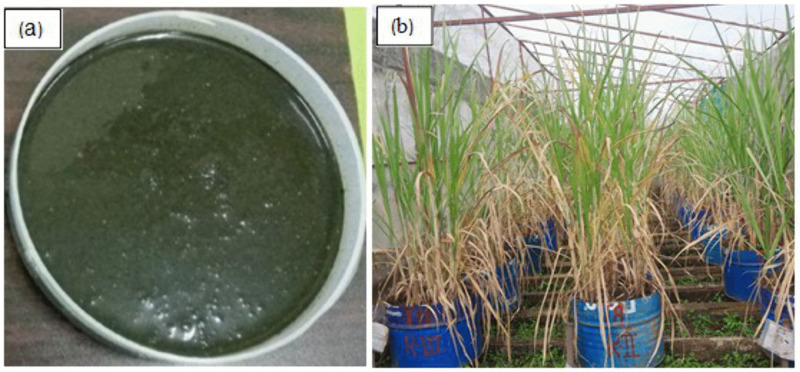
Sample picture of (a) the prepared organic fertilizer and (b) the pot experiment.

Pictures of the organic fertilizer and the sugarcane plants are shown in Fig 2. The prepared organic fertilizer (Fig 2a) was a homogenous liquid with an olive-brown color and an unpleasant odor. Liquid fertilizers provide readily available nutrients that can easily be absorbed by plants. However, they are also more prone to leaching into the environment. The sugarcane was grown in a greenhouse using pot experiments, as shown in Fig 2b below.

### 3.2. Effect of organic fertilizer application

#### 3.2.1. Effect of organic fertilizer on sugarcane growth and yield.

There was a significant difference among treatments on millable stalk count (Table 4). The highest millable stalk count was recorded from the conventional treatment. However, applications of 2.5, 5 and 7.5 tonnes ha^-1^ organic fertilizer with 50% recommended fertilizer rates were statically at par with the conventional treatments on millable stalk count. In agreement with this result, Sharma et al. found a higher number of millable cane following the application of press and mud combined with urea at a 1:1 ratio [48]. They also noted that application of organic waste increases the availability of macro and micro nutrients to crops and supplies carbon to beneficial soil microorganisms, thereby promoting decomposition and nutrient transformation process.

**Table 4:** Effect of organic fertilizer on sugarcane growth and yield.

Treatment	Millable stalk count	Leaf length (cm)	Leaf width (cm)	Number of internodes	Plant height (cm)	Internode diameter (cm)	Stalk weight (kg pot^-1^)
1	10.00ab	120.87	2.77ab	11.90	69.71	1.87abc	2.33a
2	11.0a	121.60	2.86ab	12.30	75.53	1.86abcd	2.33a
3	5.67e	117.13	2.19 cd	13.77	71.12	1.80abcde	1.17c
4	7.00de	110.73	2.10d	11.37	55.46	1.68de	1.17c
5	6.67de	112.40	2.06d	11.44	48.32	1.67e	1.00c
6	7.33cde	117.67	2.52bcd	12.52	67.86	1.81abcde	1.33c
7	9.33abc	116..07	2.66abc	12.61	66.20	1.89ab	2.17a
8	8.67bcd	111.13	2.27 cd	12.03	63.91	1.69cde	1.5bc
9	9.33abc	120.87	3.02a	12.09	72.41	1.94a	2.17a
10	7.33cde	117.60	2.25 cd	13.29	74.06	1.72bcde	1.50bc
11	10.67ab	117.67	2.45bcd	12.23	67.50	1.80abcde	2.00ab
Mean	8.45	116.77	2.47	12.32	66.55	1.81	1.70
CV	14.06	5.86	10.17	11.11	14.50	5.31	18.49
LSD	**	NS	**	NS	NS	*	**

There was a significant difference among treatments on leaf width (Table 4). The highest leaf width was observed with the application of 5 tonnes ha^-1^ organic fertilizer combined with 50% recommended fertilizer rate, followed by the conventional treatment. Similarly, treatments affected internode diameter (Table 4). The greatest internode diameter was obtained with the application of 5 tonnes ha^-1^ organic fertilizer combined with 50% recommended fertilizer. However, it was statistically comparable with the conventional treatment. Srivastava et al. also reported that the application of manure combined with recommended NPK resulted in significant improvement in sugarcane growth and yield attributes compared to the sole use of chemical fertilizer [49].

Treatments differ significantly in stalk weight (Table 4). The highest stalk weight was recorded under the conventional treatment, followed by the application of 2.5, 5 and 7.5 tonnes ha^-1^ organic fertilizer combined with 50% recommended fertilizer. Overall, the combined application of 2.5, 5 and 7.5 tonnes ha^-1^ organic fertilizer with 50% recommended fertilizer showed satisfactory performance and was statically comparable to the conventional treatment in terms of sugarcane growth and yield. However, sole applications of organic fertilizer at all rates were inferior. Consistent with this finding, Feder reported that pig slurry and sugarcane vinasse, with or without mulch, did not produce higher cane yield than chemical fertilizer alone [50]. They noted that crops respond to organic waste products alone only when nitrogen inputs are relatively high, and that optimization of the mineral supplementation allows comparable yields to be achieved.

#### 3.2.2. Effect of organic fertilizer on sugarcane foliar nutrient concentration and juice quality.

The foliar N concentration levels of the experiment ranged from 1.21–1.57% with average value of 1.33%. Even if there was no significant difference between treatments as the level of organic fertilizer increased, the foliar N concentration showed an increasing trend (Table 5). However, according to McCray and Mylavarapu the optimum N range for sugarcane is from 2.0–2.6% with a critical value of 1.8% [33]. The foliar N concentration across all treatments in the experiment was lower than the optimum and critical values. The possible reason for this could be the low level of N in the fertilizers.

**Table 5:** Effect of organic fertilizer on sugarcane foliar nutrient concentration and juice quality.

Treatment	Foliar N	Foliar P	Juice Phosphate (mg/kg)	Brix (%)	Pol (%)	Purity (%)	Recoverable sugar (%)
1	1.21	0.75abcd	102.67bc	19.10a	17.63ab	92.41ab	12.45ab
2	1.43	0.72abcd	78.67c	19.26a	17.96a	93.25a	12.79a
3	1.23	0.70abcd	221.67a	15.98b	14.64 cd	90.31bc	10.17d
4	1.21	0.77abc	223.30a	17.56ab	15.77bcd	89.83c	10.90 cd
5	1.27	0.75abcd	217.00a	18.82b	14.45d	91.25abc	10.12d
6	1.24	0.78a	154.00ab	18.52a	17.15ab	92.61ab	12.14abc
7	1.23	0.773ab	154.67ab	19.25a	17.77ab	92.28ab	12.55ab
8	1.39	0.69 cd	172.00ab	17.09ab	15.83bcd	92.61ab	11.21bcd
9	1.49	0.68d	173.00ab	18.73a	17.39ab	92.87a	12.34abc
10	1.40	0.697bcd	184.67a	17.84ab	16.58abc	92.88a	11.77abc
11	1.57	0.71abcd	148.00abc	17.74ab	16.12abcd	90.94abc	11.24bcd
Mean	1.33	0.73	166.33	18.17	16.48	91.93	11.61
CV	13.80	5.55	23.44	6.76	6.61	1.42	6.62
LSD	NS	*	**	*	**	*	**

Foliar P concentration in sugarcane differed significantly among treatments, ranging from 0.68–0.78% with average value of 0.73% (Table 5). The optimum P range for sugarcane has been reported as 0.22–0. 30%, with a critical value of 0.19% [33]. Therefore, foliar P concentration under all treatments was above the critical level. Moreover, highly significant differences were observed among treatments in juice phosphate content (Table 5). All treatments that received organic fertilizer alone or in combination with chemical fertilizer showed better content of juice phosphate as compared to chemical fertilizers alone. The addition of organic fertilized to chemical fertilizer increased sugarcane juice phosphate content by 50%, while the sole application of organic fertilizer resulted in the highest juice phosphate content among all treatments. These results suggest that combining chemical fertilizer with organic fertilizer may reduce phosphate uptake by the crop compared with the sole application of organic fertilizer. This effect may be attributed to the higher organic carbon content in the organic fertilizer, which promotes soil microbial growth and enhances phosphorus uptake efficiency.

Highly significant differences among treatments were observed for pol (%) cane and recoverable sugar, while significant differences were noted for brix (%) and purity (%) (Table 5). For all juice quality parameters brix, pol, purity and recoverable sugar, the highest value were recorded under the conventional treatment. However, these were statistically comparable to all rates of the combined application of organic and chemical fertilizer. In contrast, the sole application of organic fertilizer was inferior for all juice quality parameters except juice phosphate. The combined application of compost and inorganic fertilizer in sugarcane cultivation has been shown to enhance stalk population, number of internodes, and cane yield [13], as well as stalk girth, stalk weight, cane yield and sugar yield [14].

### 3.3. Effect of organic fertilizer application on soil physicochemical properties

#### 3.3.1. Physicochemical properties of the soil before harvest.

The experimental soil had 18.67% sand, 29.33% silt and 52% clay (Table 6), hence according to the USDA textural triangle the experimental soil was classified into a clay textural group. Blackburn noted that sugarcane can grow on soils varying in texture from light sands to heavy clays [51]. Moreover, the pH of the experimental soils was 7.77 while its EC was 0.11 dS/m (Table 6). According to FAO, sugarcane grows well in soils with pH values ranging from 5.0–8.0 while according to Landon sugarcane maintains 100% yield potential with EC levels up to 1.7 dSm^-1^ [31,52].

**Table 6:** Physicochemical properties of the soil before harvest.

Sample	Sand (%)	Silt (%)	Clay (%)	Textural Class	pH (1:5)	EC dS/m (1:5)	OC (%)	Total N (%)	Available P (mg/kg)
1	19	28	53	Clay	7.65	0.10	1.87	0.20	12.77
2	18	30	52	Clay	7.8	0.09	1.61	0.15	16.54
3	19	30	51	Clay	7.85	0.15	1.07	0.14	15.41
Mean	18.67	29.33	52	Clay	7.77	0.11	1.52	0.16	14.91

The available P content of the experimental soil was 14.91 mg/kg (Table 6), it was on the margin line for likely response of P fertilizer. Because IFA reported that there could be likelihood responses of sugarcane if the soil available P value is below 14 mg/kg [53]. The experimental soil had an organic carbon content of 1.52% and total nitrogen content of 0.16% (Table 6). According to the Landon rating scale organic carbon content of a soil less than 2% is considered as very low [31] and fertilizer recommendation guideline indicated that, soil total N value between 0.091 and 0.18% is low [32]. Therefore, for optimum growth and yield of sugarcane, application of organic matter and chemical fertilizers was crucial for the experimental soil.

#### 3.3.2. Physicochemical properties of the soil after harvest.

There was no significant difference between treatments on soil pH and EC due to the application of organic fertilizer for sugarcane (Table 7). At the same time, the values of pH and EC before planting and after harvest had no difference at all. From this, it can be concluded that the organic fertilizer will not have any adverse effect on soil reaction (acidity and alkalinity) as well as salinity. Even if there was no significant difference between treatments on soil organic carbon (OC) content due to the application of organic fertilizer (Table 7), on average there was a sharp decline from 1.52% OC of before planting (Table 6) to 0.90% OC of after harvest (Table 7). In agreement with this result, Adiku *et al* found OC depletion caused by crop cultivation without organic matter incorporation [54].

**Table 7:** Physicochemical properties of the experimental soils after harvest.

Treatments	pH (1:5)	EC dS/m (1:5)	OC (%)	Total N (%)	Available P (mg/kg)
1	7.92	0.10	0.90	0.13bc	3.15d
2	7.83	0.09	0.94	0.12bc	3.61 cd
3	7.74	0.08	0.86	0.14bc	5.34 cd
4	7.83	0.09	0.87	0.15b	5.33 cd
5	7.72	0.08	0.89	0.13bc	3.99 cd
6	7.71	0.10	0.98	0.18a	5.81bc
7	7.75	0.09	0.86	0.12bc	4.09 cd
8	7.72	0.10	0.89	0.12bc	13.43a
9	7.76	0.08	0.92	0.14bc	5.51c
10	7.71	0.11	0.87	0.14bc	4.52 cd
11	7.77	0.10	0.90	0.14bc	7.68b
Mean	7.77	0.09	0.90	0.14	5.68
CV	1.69	17.14	8.07	9.65	18.02
LSD	NS	NS	NS	*	**

Application of organic fertilizer for sugarcane showed significant difference between treatment on total N and highly significant difference on available P (Table 7). The highest soil total N was recorded from the combination of 25% recommended chemical fertilizer with 2.5 tonnes ha^-1^ organic fertilizer. Similarly, the highest soil available P was recorded from the combination of 25% recommended chemical fertilizer with 5 tonnes ha^-1^ organic fertilizer. However, there was a slight decline on the average soil total N content (from 0.16 to 0.14%) and sharp decline on the available P content (from 14.91 to 5.68 mg/kg) of the experimental soil as compared to the results of before planting (Tables 6 and 7). This may be due to the fact that sugarcane is a heavy feeder crop with its larger biomass utilizing N and P to a greater extent thereby causing soil N and P depletion.

## 4. Conclusion

Organic fertilizer was successfully prepared from slaughterhouse meat waste and evaluated for its chemical composition, physicochemical properties and microbial activities. The analyses revealed that the fertilizer contained low levels of essential nutrients such as N, P and K. To meet crop nutritional requirements, the fertilizer should either be enriched with additional nutrients or applied in higher quantities. Most of the measured physicochemical properties suggest that the fertilizer is suitable for sugarcane cultivation. Furthermore, the presence of beneficial microbial species such as *Lysinibacillus* sphaericus, *Bacillus simplex, Bacillus cereus, Kocuria varians,* and *Microascus* potential to enhance soil health, promote nutrient cycling, and support sustainable agriculture practice. The combined application of 2.5, 5 and 7.5 tonnes ha^-1^ organic fertilizer with 50% of the recommended chemical fertilizer resulted in better performance across most measured parameters than the same organic fertilizer rates combined with either 25% or 75% of the recommended chemical fertilizer. Notably, the application of 5 tonnes ha^-1^ organic fertilizer combined with 50% of the recommended chemical fertilizer achieved performance comparable to that of the full recommended chemical fertilizer rate for nearly all measured parameters. However, solo application of the prepared fertilizer was inferior for all measured parameters except juice phosphate. These findings suggest that reducing chemical fertilizer input by 50% and supplementing with organic fertilizer could maintain sugarcane growth, yield and juice quality, providing a more sustainable and cost effective alternative. Hence, to develop a compressive recommendation, it is critical to verify the pot experiment results under field conditions and to conduct economic feasibility analyses.
